# Happy Birthday, Dear Journal!

**DOI:** 10.1055/a-2519-5062

**Published:** 2025-03-27

**Authors:** Jörg Bahm

**Affiliations:** 1 Division for Plexus Surgery, Department of Plastic, Hand and Burn Surgery, University Hospital Aachen Germany

**Figure:**
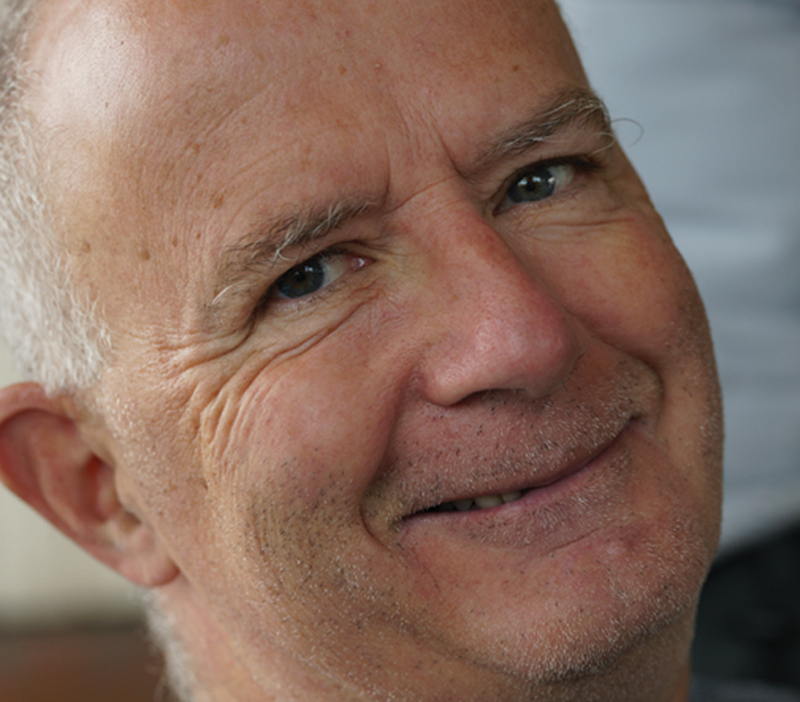
Jörg Bahm, MD, PhD

On September 29, 2006, Rahul Nath from Houston, Texas wrote the inaugural editorial for the Journal of Brachial Plexus and Peripheral Nerve Injury (JBPPNI), published online by BioMed Central.

In February 2007, I joined the editorial board and later became the Editor-in-Chief.

December 2015 marked a change, as Thieme kindly accepted to become the new publisher, which allowed us to join a very professional team with outstanding technical capacities and a huge platform to increase the journal's visibility and high-quality production services.

In the first month of 2025, we now suddenly stand with an already 20 ^th^ anniversary and an established online journal which in 2024 had 46,367 (over 46 thousand !!!) article downloads, which means on average 125 articles per day. 1

Moreover, beside regular citation in major indexing and abstracting services, the journal actually holds a solid impact factor of 1.1. in 2023, 2 a mark many academic contributors need to publish their research with us, to make it valuable for their academic career.

So far concerning the technical aspect, but what about content, our readers and advisership?

Submissions were received from over 40 countries, regardless the economic income (the Thieme policy on APC discounts and waivers continuously allows us to counteract the bias of publication costs for authors from low- and middle-income countries); and the potential reviewer list is still growing. The increase in downloads in the last 10 years is 13-fold, a huge progression! 3

Not only are we covering all aspects of peripheral nerve surgery and related research, by publishing both original articles and reviews, but the articles and authors of the last 20-year feature a historical review of how and by whom the development of this particular interdisciplinary surgical subspeciality developed and continues to grow. 4

Scrolling the issues of the past years, with some five articles per year, gives a realistic overview of what happens in several clinical fields, where basic research on peripheral nerve regeneration and animal models stands and what could be further developments. The Cited Half-life is nearly 12 years, meaning articles published in JBPPNI remain relevant to the community for over a decade. 2

We still may not compete with the huge specialty journals, giants in neurosurgery, plastic, or orthopaedic surgery. But we achieved to become an innovative and high-quality platform for those colleagues who are dealing regularly with peripheral nerve issues, including all surgical techniques.

It’s a reason to be proud about our reliable publisher Thieme, who continues to offer an online platform of high quality, professional assistance to the editor, reviewers, and all authors—thereby contributing actively to a sound promotion of valid knowledge and scientific data.

It’s thereby a moment to express our gratefulness to the publisher and the publishing team, to all dedicated reviewers and authors who have continued to trust us over these past 20 years. Thank you all for letting JBPPNI grow an adult and please stay with us in the forthcoming years to maintain the same appreciation and pleasure of exchanging valuable information, share ideas, techniques, and results, and further develop the field of peripheral nerve surgery with great passion!

Aachen, January 7, 2025 Jörg Bahm MD, PhD, Editor-in-Chief JBPPNI.
